# Eating Time as a Genetic Indicator of Methane Emissions and Feed Efficiency in Australian Maternal Composite Sheep

**DOI:** 10.3389/fgene.2022.883520

**Published:** 2022-05-11

**Authors:** Boris J. Sepulveda, Stephanie K. Muir, Sunduimijid Bolormaa, Matthew I. Knight, Ralph Behrendt, Iona M. MacLeod, Jennie E. Pryce, Hans D. Daetwyler

**Affiliations:** ^1^ Agriculture Victoria, AgriBio, Centre for AgriBioscience, Bundoora, VIC, Australia; ^2^ School of Applied Systems Biology, La Trobe University, Bundoora, VIC, Australia; ^3^ Agriculture Victoria, Hamilton Centre, Hamilton, VIC, Australia

**Keywords:** eating time, methane production, methane yield, residual methane, feed efficiency

## Abstract

Previous studies have shown reduced enteric methane emissions (ME) and residual feed intake (RFI) through the application of genomic selection in ruminants. The objective of this study was to evaluate feeding behaviour traits as genetic indicators for ME and RFI in Australian Maternal Composite ewes using data from an automated feed intake facility. The feeding behaviour traits evaluated were the amount of time spent eating per day (eating time; ETD; min/day) and per visit (eating time per event; ETE; min/event), daily number of events (DNE), event feed intake (EFI; g/event) and eating rate (ER; g/min). Genotypes and phenotypes of 445 ewes at three different ages (post-weaning, hogget, and adult) were used to estimate the heritability of ME, RFI, and the feeding behaviour traits using univariate genomic best linear unbiased prediction models. Multivariate models were used to estimate the correlations between these traits and within each trait at different ages. The response to selection was evaluated for ME and RFI with direct selection models and indirect models with ETE as an indicator trait, as this behaviour trait was a promising indicator based on heritability and genetic correlations. Heritabilities were between 0.12 and 0.18 for ME and RFI, and between 0.29 and 0.47 for the eating behaviour traits. In our data, selecting for more efficient animals (low RFI) would lead to higher methane emissions per day and per kg of dry matter intake. Selecting for more ETE also improves feed efficiency but results in more methane per day and per kg dry matter intake. Based on our results, ETE could be evaluated as an indicator trait for ME and RFI under an index approach that allows simultaneous selection for improvement in emissions and feed efficiency. Selecting for ETE may have a tremendous impact on the industry, as it may be easier and cheaper to obtain than feed intake and ME data. As the data were collected using individual feeding units, the findings on this research should be validated under grazing conditions.

## Introduction

Two major challenges for the Australian sheep industry are to reduce enteric methane emissions and to improve feed efficiency.

Feed efficiency in sheep is a trait of socio-economic importance. The efficient use of feed by animals is vital in providing animal protein to an expanding human population, especially on land that cannot be used to produce human food. Feed is the highest production cost in the sheep industry, and for that reason, improving feed efficiency could increase profitability. Residual feed intake (RFI) is an accepted measure of feed efficiency that estimates the difference between the actual and predicted daily dry matter intake (DMI) (Koch et al., 1963; Fogarty et al., 2006). RFI is predicted using a linear regression of actual feed intake on the variables that explain the energy used for maintenance, growth, and production. Animals with negative RFI values consume less feed to produce the same amount of meat than animals with positive RFI values and are therefore considered more efficient. There are limited studies on the genetics of RFI in sheep, but it has been demonstrated that it is heritable and selection for RFI would not compromise other production traits (Tortereau et al., 2020).

The reduction of methane emissions from sheep production is an important goal. Australia is committed to reducing greenhouse gas emissions (NDC, 2020) and decreasing methane emissions is critical for achieving this target, as methane is one of the main contributors to global warming. Agriculture is the most significant anthropogenic methane source in the global methane budget, and ruminants contribute to it through enteric fermentation and manure management (Saunois et al., 2019). Sheep emit methane as a byproduct of rumen fermentation during digestion. In addition to its environmental impact, methane emissions represent a waste of 6–10% of gross energy intake that could be used for productivity (Pinares-Patiño et al., 2013). Methane emission traits include methane production (MeP), methane yield (MeY = MeP/DMI), and residual methane production (RMP = actual MeP—predicted MeP) (de Haas et al., 2017).

Feeding behaviour is the behaviour of animals related to access and consumption of food. These traits could be of interest as indicators for methane emission and residual feed intake. Feeding behaviour traits include eating time (i.e., the amount of time spent eating), number and size of eating events and eating rate. Feeding behaviour traits, methane emissions, and RFI have been shown to be under partial genetic control (Table 1). Phenotypic and genetic correlations of feeding behaviour traits with RFI have been reported in sheep (Cammack et al., 2005; Muir et al., 2018; Marie-Etancelin et al., 2019) and methane emissions are associated with digesta kinetics (Pinares-Patiño et al., 2003b), which could reflect changes in feeding behaviour. Furthermore, feeding behaviour records could potentially be collected on commercial farms with the use of wearable sensors, a promising technology for sheep (Fogarty et al., 2020) that is already used in cattle (Beauchemin, 2018). These feeding behaviour records would significantly increase the amount of data for genetic analysis compared with the number of animals that are required to be measured for feed intake and methane production. Therefore, feeding behaviour traits are of potential interest as indicators for feed efficiency and methane emissions.

**TABLE 1:** Heritability reported in literature of methane production (MeP), methane yield (MeY), residual methane production (RMP), dry matter intake (DMI), residual feed intake (RFI), metabolic mid-weight (MMWT), average daily gain (ADG), eating time per day (ETD), eating time per eating event (ETE), daily number of events (DNE), event feed intake (EFI), and eating rate (ER). * PAC: portable accumulation chamber, RC: respiration chambers.

Trait	Heritability in literature	References
MeP	*PAC: 0.10 to 0.19	PAC: Goopy et al. (2016); Paganoni et al. (2017); Jonker et al. (2018b)
	*RC: 0.23 to 0.31	RC: Robinson et al. (2010); Pinares-Patiño et al. (2013); Jonker et al. (2018b); Jonker et al. (2019); Rowe et al. (2019)
MeY	0.13 to 0.15	Pinares-Patiño et al. (2013); Jonker et al. (2018b); Jonker et al. (2019); Rowe et al. (2019)
RMP	0.13 to 0.19 in cattle	Herd et al. (2014); Hayes et al. (2015); de Haas et al. (2016); Manzanilla-Pech et al. (2016); Manzanilla-Pech et al. (2021)
DMI	0.25 to 0.49	François et al. (2002); Leymaster et al. (2002); Snowder and Van Vleck, (2003); Cammack et al. (2005); Fogarty et al. (2006); Fogarty et al. (2009); Paganoni et al. (2017); Rowe et al. (2019); Tortereau et al. (2020)
RFI	Discounting maintenance and growth: 0.11 to 0.38	Discounting maintenance and growth: Snowder and Van Vleck, (2003); Cammack et al. (2005); Paganoni et al. (2017); Hess et al. (2019)
	Discounting maintenance, growth, body backfat and muscle depth: 0.30 to 0.45	Discounting maintenance, growth, body backfat and muscle depth: François et al. (2002); Tortereau et al. (2020)
MMWT	0.20 to 0.50	Fogarty, (1995); Snowder and Van Vleck, (2003); Fogarty et al. (2009); Jonker et al. (2018b); Hess et al. (2019); Rowe et al. (2019)
ADG	0.18 to 0.50	Shrestha et al. (1985); Shrestha et al. (1986); Mousa et al. (1999); Bibé et al. (2002); François et al. (2002); Leymaster et al. (2002); Snowder and Van Vleck, (2003); Cammack et al. (2005); Tortereau et al. (2020).
ETD	In sheep: 0.24 to 0.36	In sheep: Leymaster et al. (2002); Cammack et al. (2005)
	In dairy heifers: 0.50	In dairy heifers: Lin et al. (2013)
ETE	0.29	Leymaster et al. (2002); Cammack et al. (2005)
DNE	In sheep: 0.33 to 0.35	In sheep: Leymaster et al. (2002); Cammack et al. (2005)
	In dairy heifers: 0.45	In dairy heifers: Lin et al. (2013)
EFI	0.33	Leymaster et al. (2002); Cammack et al. (2005)
ER	In dairy heifers: 0.46	In dairy heifers: Lin et al. (2013)

Genomic selection has generated permanent and cumulative genetic change over generations to reduce methane emissions in sheep (Rowe et al., 2019) and has the potential to improvefeed efficiency. Genomic selection could be beneficial to improve methane emissions and feed intake because measurements of these traits are difficult to obtain in commercial farms, as they are time-consuming and expensive to measure, or require specialist equipment. The genomic prediction accuracy for MeP and MeY has been reported as moderate in sheep and cattle (Rowe et al., 2014; Hayes et al., 2015); while for RFI in cattle accuracy varies from low to moderate (Pryce et al., 2015; Li et al., 2020). The accuracy of genomic breeding values for daily dry matter intake was also reported in dairy and beef cattle as moderate (Bolormaa et al., 2013; Silva et al., 2016). Eating time and daily number of eating events have moderate heritabilities (Leymaster et al., 2002; Cammack et al., 2005).

The accuracy of genomic breeding values partially depends on measuring the goal and indicator traits under environmental and management conditions that are as similar as possible to commercial production systems. In the case of the Australian Maternal Composite sheep, the commercial conditions include grazing pastures that vary in quantity and quality throughout the year. Under these conditions, the relationship between methane emissions, feed efficiency and feeding behaviour is expected to be more complex than under controlled environments. For instance, sheep under grazing conditions may show variation in their ability to walk to obtain enough food and water. However, measuring methane emissions, feed intake and feeding behaviour traits under grazing is cost-prohibitive and technically not possible at the moment. As an alternative, measuring these traits under controlled conditions is feasible and can help to elucidate the heritability of the traits and their correlations, in addition to developing genomic predictions. However, it is advisable to validate the genomic predictions under commercial conditions before implementing a genomic selection program.

The objective of this study was to evaluate the feeding behaviour traits associated to the amount of time spent eating per day (eating time; ETD) and per visit (eating time per event; ETE), daily number of events (DNE), event feed intake (EFI; g/event) and eating rate (ER; g/min) as genetic indicators for MeP, MeY, RMP, and RFI in Australian Maternal Composite ewes at post-weaning (PW), hogget, and adult ages using data from an automated feed intake facility.

## Materials and Methods

### Animals and Genotypes

Data were available on 445 Australian Maternal Composite ewes, a popular composite breed in Australia (Muir et al., 2020a). Of these ewes, 251 were born in 2013 and 194 in 2014. Fifteen, 296, and 34 ewes were daughters of Border Leicester, Coopworth, and White Suffolk sires, respectively, while the sire of 120 ewes was unknown. Dams were all Maternal Composite. From the 2013 born ewes, 81 were measured at PW, 195 at hogget, and 218 at adult stages. From the 2014 born ewes, 193 were measured at PW and 189 at hogget ages. The ages of the animals at the beginning of the test were 313 ± 14, 534 ± 19, and 858 ± 23 days old at PW, hogget and adult ages, respectively. From the 2013 and 2014 born ewes, 12 and 137 were pregnant at PW and had 108 ± 9 and 102 ± 8 days of pregnancy at the end of the test, respectively. No ewes were pregnant at hogget or adult ages during the test. No animals were lactating during the test.

The animals were genotyped with low-density [”12K” (12,785, *n* = 165) and “15K” (15,000, *n* = 110)], and medium-density [”50K” (54,241, *n* = 190)] single nucleotide polymorphism (SNP) chips. SNP were excluded if the call rate per SNP [the proportion of SNP genotypes that have a GC (Illumina GenCall) score above 0.6] was less than 0.9. Furthermore, if the average call rate per individual was less than 0.9, those animals were removed from the SNP data. After applying this quality control procedure, 9,325, 9,190, and 34,770 SNP remained for the 12K, 15K and 50K SNP chips, respectively. Subsequently, the imputation of sporadic missing genotypes within each SNP array was performed using FImpute (Sargolzaei et al., 2014). The imputation from low-density to 50K SNPs was carried out using a reference population of 1,933 animals from multiple breeds with 38,378 SNPs (Bolormaa et al., 2019). A genomic relationship matrix (**GRM**) was constructed with the imputed genotypes of the 445 Australian Maternal Composite ewes using the function Gmatrix of the R package AGHmatrix (Amadeu et al., 2016) using the method of Yang et al. (2010), and a principal component analysis on the **GRM** was also completed.

### Environment and Traits

Five feed intake experiments were conducted between 2014 and 2016 (Table 2). Feed intake was measured for individual sheep housed in groups in the automated feed intake facility at Agriculture Victoria, Hamilton, Victoria (Australia) (Muir et al., 2020b). Animals were fed with a cereal straw-based pelleted ration available *ad libitum* (24 h per day). The pellets had 9-mm in diameter and their composition was 40% cereal straw, 20% cereal grain (barley or wheat), 15% legume grains (beans, lupins or lentils), 15% oat hulls, 7% almond hulls, 1% lime, 1.5% bentonite, 0.5% gypsum. Samples of pellets were analysed using near-infrared spectroscopy. The methodological details on the pellets analysis are described by Muir et al. (2018). Pellets had 65 (± 2.4 SD) % digestibility, 9.8 (± 1.6) % crude protein (CP), 48 (± 3.18) % neutral detergent fibre (NDF), and 9.6 (± 0.58) MJ of metabolisable energy per kg of dry matter (DM).

**TABLE 2:** Feed experiments details on post-weaning, hogget, and adult Australian Maternal Composite sheep. YOB: Year of birth of animals; Animals: Number of animals in the experiment; Animals by pen: Mean number of animals by pen; Duration: test duration (days).

Experiment	YOB	Start/End of experiment	Animals	Age stage	Animals by Pen	Duration (d)
1	2013	2014-07-03/2014-08-29	81	Post-weaning	8.1	57
2	2013	2015-02-04/2015-03-20	195	Hogget	19.5	44
3	2013	2015-12-21/2016-01-22	218	Adult	21.8	32
4	2014	2015-06-22/2015-08-12	193	Post-weaning	19.3	51
5	2014	2016-02-08/2016-03-18	189	Hogget	18.9	39

Animals were spread across 10 pens each pen was equipped with two automated feeders and there was continuous access to water. After an adaptation period to the diet of 10–14 days, feed intake was measured for a total of 53 (±3), 42 (±3) and, 32 (±0) days for PW, hogget, and adult stages, respectively. The experiments were planned for a minimum of 42 days, but 2013-born adults and 2014-born hoggets were meadured for 32 and 39 days, respectively, (Table 2) to fit around management requirements (e.g., timing of mating on the farm). However, there is evidence that the feed intake of sheep can be estimated accurately in less than 35 days (Macleay et al., 2016; Amarilho-Silveira et al., 2022), with intake stabilising by about 21 days after adaptation (Johnson et al., 2017). Amarilho-Silveira et al. (2022) reported correlations greater than 0.96 between feed intake measured at 21 and 42 days and correlations greater than 0.90 for the RFI estimated with these records.

Feed intake was recorded automatically with a specific radio frequency identification tag each time an individual animal access the feed units (eating event). The automated feeders created and stored individual animal feeding events in addition to refilling of feed bins between feed events to 1,000 g—meaning that each animal had access to 1,000 g of feed at each visit. There was no limit on the number of feeder visits (eating events), or the time spent eating per visit (eating time per event; ETE) and, hence, the total feed consumption was not restricted. The automated feed system, operation and accuracy is described in detail by Muir et al. (2020b). Pens had dirt floors, shed roof line of ∼7.4 m high in the centre and ∼4 m high at the outside edge, and a close shed in the northern side to partially protect animals from wind. The east, west and south sheds were open, and therefore animals would be experiencing close to ambient temperatures, although clearly without any sun or rain. Each pen was 4.8 m wide × 18 m long (∼ 86 m^2^), of which ∼80 m^2^ was free space for the sheep. No more than 24 ewes were included in each pen (3.33 m^2^ per sheep at maximum density), and there was a balanced distribution of sires and ewe live weights across pens. Although there were different numbers of sheep per feeder in each feeding period, the occupancy of the feeders in all periods was low enough to allow sheep to consume feed *ad libitum*, meaning that between animal interactions were unlikely to have affected eating rate substantially. Each pen had approximately the same number of animals in each experiment, but animals were not necessarily assigned to the same pen in different experiments at different ages.

The design of the feeders made impossible for sheep to push each other out of the feeder because once an animal entered the feed race, an electromagnetic lock on the gate behind them was activated and kept lock while the animal was eating, and no other sheep was able to access the feeder or got close enough to the eating individual to push them out of the feeder. (Muir et al., 2020b). Live weights were measured three times weekly during the feed intake experiment and used to estimate the average daily gain (ADG).

Residual Feed Intake was calculated using the following model:

$$DMI=mean+b_{1}MMWT+b_{2}ADG+b_{3}EXP+b_{4}PEN:EXP+b_{5}AGE+b_{6}Pregnancy+b_{7}Pregnancy\;Scan+RFI,$$ (1)

Where $b_{1}$ - $\;b_{7}$ are partial regression coefficients; DMI is measured daily dry matter intake; MMWT is metabolic mid-test weight, an estimation of the maintenance requirements of an animal, obtained as the average weight of the animal during a feeding test to the power of 0.75; EXP is the feed intake experiment (*n* = 5); PEN:EXP is the pen where animals were kept within each experiment nested into EXP (45 levels), AGE is the age in days at the start of the experiment, Pregnancy is days of pregnancy at the end of the experiment, and Pregnancy Scan is the number of lambs in pregnancy scan. RFI is the residual error of the equation and represents the intake after accounting for energy sinks. In this case, these sinks are the energy used for maintenance (MMWT) and growth (ADG) adjusted for EXP, PEN, and AGE.

Feeding behaviour records were also obtained during feeding experiments using the automated feeding system (Muir et al., 2020b). Sheep initiate the feeding event by entering the feeder and coming within range of a proximity sensor and a radio frequency identification (RFID) reader, after which the flap covering a trough mounted on scales opens. Once a sheep finishes eating and leaves the feeder, exiting the range of the proximity sensor and RFID reader, the flap and feed event closes (Muir et al., 2020b). The time the animal was in the feeder from the start to the end of the feeding event was considered eating time. This period includes the time spent consuming feed and any periods when the animal is still within the range of the proximity sensor and radio frequency identification (RFID) reader whilst the flap covering the trough is open. We then calculated the eating time (ET) per day (ETD; min/d) and per eating event (ETE; min/event). The daily number of events (DNE), event feed intake (EFI; g/event) and eating rate (ER; g/min) were also calculated to investigate the effect that selection on ET would have on other feeding behaviour traits.

Methane emissions were recorded twice during each feed intake experiment with the portable accumulation chamber (PAC) technique described by Goopy et al. (2011). Twenty-four chambers, each with an internal volume of 819L, were used to record methane emissions. The first methane measurement was performed approximately 4 weeks from the start of the experiment with a second measurement approximately 10 days later. It is known that the time since the last meal affects methane emissions (Muir et al., 2021), and therefore, the sampling procedure was designed to minimise the effect of time of day. A pen of ewes sampled in the morning for the first measurement was not sampled at the same time for the second measurement. Each sheep was placed in a chamber within approximately 30 min after being removed from access to feed and the concentration of methane (ppm) was measured at 15-min intervals for 45 min. Further details on how methane was recorded are provided by Muir et al. (2020a). MeP (L/d) was estimated with the methodology of Jonker et al. (2020) as:

$$MeP\left(\frac{L}{d}\right)=\left(\frac{\left(\mathrm{CH}4\left(ppm\right)-background\left(ppm\right)\right)}{100,000}\right)*\left(\frac{air\;volume\;in\;PAC\;\left(L\right)}{time\;in\;PAC\;\left(min\right)}\right)*60*24$$ (2)

Where background is the CH4 concentration at time 0; air volume in PAC = PAC volume (L) - body volume of the sheep (assumed 1 kg body weight = 1.0 1.01 L); and 60 and 24 are minutes and hours, respectively. MeY (MeP/DMI) and RMP were also calculated for each animal. RMP was estimated with the model:

$$MeP=mean+b_{1}DMI+b_{2}MMWT+b_{3}ADG+b_{4}EXP+b_{5}PEN:EXP+b_{6}AGE+b_{7}Pregnancy+b_{8}Pregnancy\;Scan+RMP,$$ (3)

Where $b_{1}$ - $b_{8}$ are partial regression coefficients, and RMP is the residual error of the equation. RMP represents MeP after accounting for the energy used for maintenance (MMWT) and growth (ADG) and adjusted by DMI, experiment, pen, age, days of pregnancy at the end of the experiment, and number of lambs in pregnancy scan. The fixed effects used to obtain RMP were the same as those for RFI but included DMI as it is known that intake is correlated with MeP. For all traits, the records four standard or over deviations from the mean were removed.

### Heritability, Correlations, Genomic Prediction Accuracy and Bias

Heritability (*h* ^*2*^), and phenotypic (*r*_*p*_) and genetic correlations (*r*_*g*_) were obtained with the R package ASReml-R (Butler et al., 2009).

Assuming that the same trait was measured at each age stages and that the effect of days of age was linear on each of these traits, heritabilities were obtained for combined age groups with univariate genomic best linear unbiased prediction (GBLUP) models of the form

y=1µ+Xβ+Zg+e, (4)

Where y is a vector of phenotypes; µ is the overall mean; β is a vector of fixed effects; g is a vector of random additive genetic effects; X and Z are the incidence matrices for β and g , respectively, and e is a vector of random residuals distributed as $N\left(0,I\sigma_{e}^{2}\right)$ . The distribution of g is $N\left(0,G\sigma_{g}^{2}\right)$ , where G is the genomic relationship matrix (VanRaden, 2008) calculated from SNP genotypes following Yang et al. (2010). **Model 1** included in β the fixed effects EXP, PEN nested into EXP,AGE, Pregnancy, Pregnancy Scan, and the first two principal components of the **GRM** (PC1 and PC2). PC1 and PC2 were included because it has been reported that genomic prediction accuracies in multi-breed sheep tends to be overestimated due to population structure, and this effect can be reduced by including principal components of the **GRM** as covariates (Daetwyler et al., 2012). **Model 1** was used for all traits except RFI and RMP. **Model 2** only included the fixed effects PC1 and PC2 and was applied to RFI and RMP because their phenotypes were obtained with the rest of fixed effects of **model 1** as regressors.

**Model 1** in the form of bivariate GBLUP was used for estimating correlations between most of the traits, with some exceptions, where **model 2** (as a bivariate GBLUP) was implemented. For example, **model 2** was used to estimate the correlations of RMP and RFI with those traits used in their estimation, because RMP and RFI are the residuals of models that already include the same fixed effects of **model 1** (except PC1 and PC2) as regressors. **Model 2** was also used for instances when there were convergence issues for **model 1**, for example, between MeY and ER.

Phenotypic and genetic correlations were estimated between different stages of age. Multivariate GBLUPs were conducted to estimate genetic correlations between PW, hogget, and adult stages to determine whether the traits could be considered the same in all ages. **Model 1** and **model 2** were used for all traits replacing the mean with its multivariate equivalent.

Genomic prediction accuracy and bias of the univariate models were estimated with a five-fold cross-validation, for this, animals were randomly assigned into five cohorts. One cohort was used as a validation set, and their phenotypic data were removed, while the remaining 4 cohorts were used to calculate the estimated breeding values (GEBVs). Except for RMP and RFI, the accuracy was estimated as the Pearson correlation between the GEBVs and phenotypes corrected for fixed effects using **model 1**, and the bias as the regression slope of these adjusted phenotypes on GEBVs. The accuracy and bias of RMP and RFI were estimated as previously described, but the phenotypes were not adjusted for the fixed effects of **model 1** because these effects were already included in the models used to obtain the phenotypes. This was repeated with every cohort as the validation set and averaged across cohorts.

### Potential Response to Selection

The response to selection on methane emissions and RFI with direct selection and indirect selection with eating time per eating event as indicator were estimated. Annual direct and indirect responses to selection on methane emission traits and RFI were predicted. The direct responses were calculated as:

$$\text{Δ}G=\frac{i*r*\sigma_{a}}{L},$$ (5)

Where i is the intensity of selection assumed as 1.5 which is equivalent to selecting the top 7% of animals to be parents; r is the accuracy of the GEBVs, assumed between 0.2 and 0.8 for all traits; $\sigma_{a}$ is the genetic standard deviation for the trait; and L is the generation interval (assumed to be 3 years). The indirect responses to selection on MeP, MeY, and RFI using ETE as the indicator trait were calculated as:

$$\text{Δ}G=\frac{i*r*\sigma_{a}*r_{g}}{L},$$ (6)

Where values for i , L and r are the same as in the direct response to selection, $\sigma_{a}$ is the genetic standard deviation for the breeding goal trait, and $r_{g}$ is the genetic correlation between the indicator and breeding goal traits estimated with **model 1**.

## Results

**FIGURE 1:**
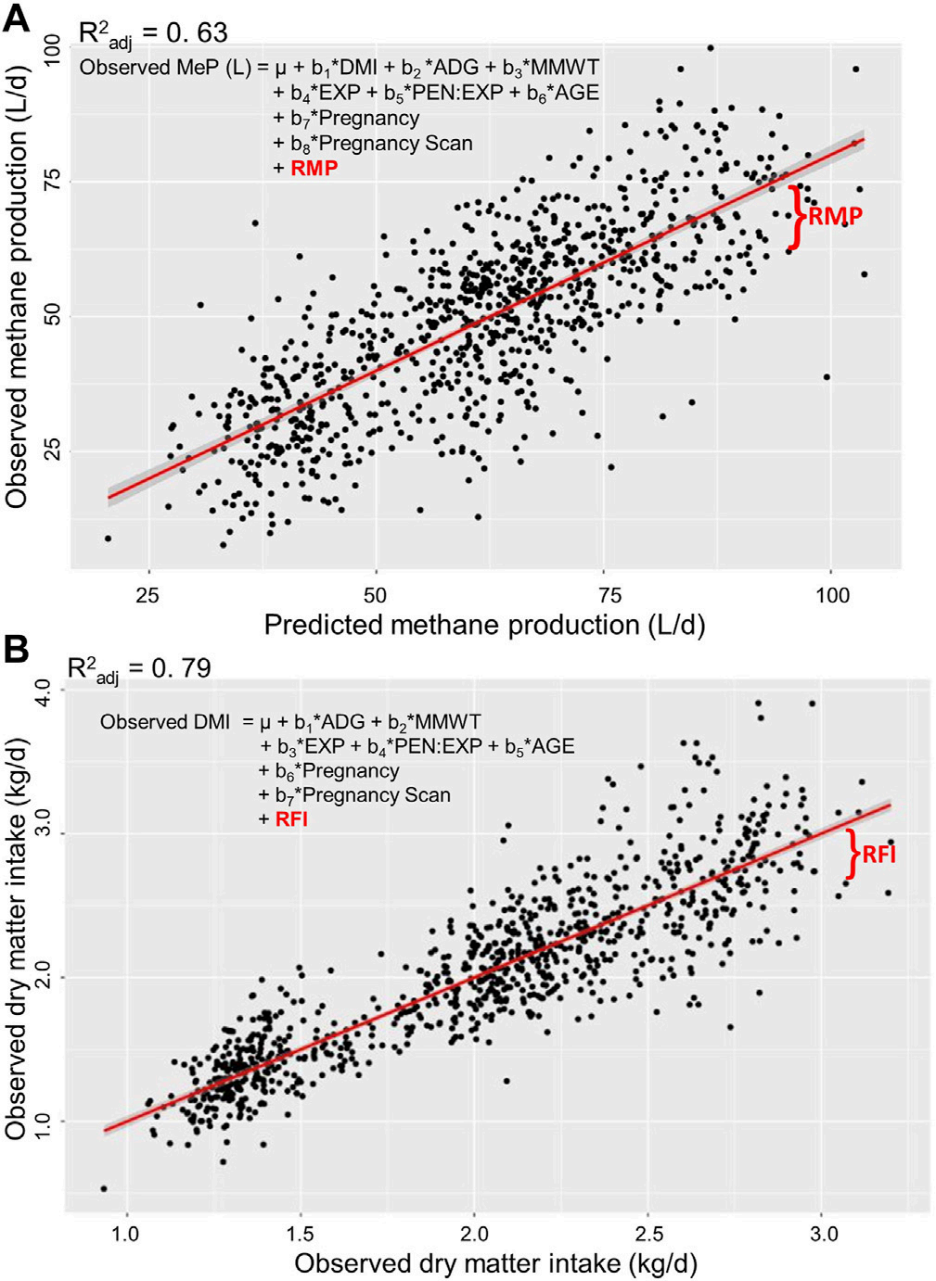
Estimation of residual traits in post-weaning, hogget, and adult Australian Maternal Composite sheep. Residual methane production [RMP; **(A)**] is the residual of observed methane production (MeP) regressed on predicted MeP. Residual feed intake RFI; **(B)** is the residual of observed daily dry matter intake (DMI) regressed on predicted DMI. Regressors: DMI, average daily gain (ADG), metabolic mid-weight (MMWT), experiment (EXP), pen nested into EXP (PEN: EXP), and age at the start of the test (AGE).

Approximately the same number of animals and records were analysed for each trait (Table 3). The coefficient of determination (*R* ^*2*^) of observed MeP regressed on predicted MeP was 0.63, and the *R* ^*2*^ of the observed DMI regressed on the predicted DMI was 0.79 (Figure 1).

**TABLE 3:** Descriptive statistics of traits in Australian Maternal Composite ewes. Traits: Methane production (MeP), methane yield (MeY), residual methane production (RMP), dry matter intake (DMI), residual feed intake (RFI), metabolic mid-weight (MMWT), average daily gain (ADG), eating time per day (ETD), eating time per eating event (ETE), daily number of eating events (DNE), event feed intake (EFI), and eating rate (ER).

Trait	Unit	n records	Total						Post-weaning					
			n	Mean	SD	Min	Max	CV	n	Mean	SD	Min	Max	CV
MeP	L/d	897	462	49.97	17.08	7.65	99.82	34.17	271	34.13	12.49	7.65	75.20	36.61
MeY	L/kg DMI	886	458	25.08	7.14	6.72	54.19	28.47	271	25.52	9.51	7.06	54.19	37.25
RMP	L/d	868	442	−0.01	10.21	−39.66	35.96	NA	271	−0.07	9.28	−24.65	35.96	NA
DMI	kg/d	895	461	2.01	0.60	0.53	3.91	29.71	275	1.35	0.24	0.53	2.16	17.65
RFI	kg/d	871	443	0.00	0.27	−0.96	1.05	NA	273	0.00	0.18	−0.59	0.51	NA
MMWT	kg	899	461	20.27	2.98	13.43	28.64	14.70	275	17.01	1.38	13.43	20.70	8.12
ADG	kg/d	902	463	0.18	0.08	−0.02	0.47	42.83	275	0.20	0.07	0.03	0.43	35.98
ETD	min	897	463	90.89	32.15	9.56	281.62	35.37	272	94.73	38.20	22.00	249.37	40.33
ETE	min	899	463	10.58	4.78	1.17	43.18	45.17	272	9.27	4.82	1.17	33.31	52.02
DNE	number	895	461	9.84	5.02	2.34	33.14	50.99	269	11.89	6.66	2.36	33.14	56.00
EFI	g of DM	902	463	245.67	112.53	22.00	639.30	45.80	275	141.22	68.86	22.00	557.52	48.76
ER	g/min	902	463	24.15	8.92	1.91	55.89	36.92	275	15.90	5.58	2.12	34.53	35.08
**Trait**	**Unit**	**n records**	**Hogget**						**Adult**					
						**n**	**Mean**	**SD**	**Min**	**Max**	**CV**	**n**	**Mean**	**SD**	**Min**	**Max**	**CV**
MeP	L/d	897	401	51.56	12.05	12.84	85.51	23.37	225	66.20	12.09	27.90	99.82	18.26
MeY	L/kg DMI	886	398	24.75	6.16	6.72	42.73	24.88	217	25.12	5.08	9.18	40.72	20.21
RMP	L/d	868	382	−0.01	10.47	−39.66	24.40	NA	215	0.05	10.87	−37.94	28.93	NA
DMI	kg/d	895	401	2.10	0.34	0.67	3.34	16.30	219	2.67	0.42	1.66	3.91	15.72
RFI	kg/d	871	382	0.00	0.25	−0.77	1.02	NA	216	0.00	0.38	−0.96	1.05	NA
MMWT	kg	899	397	20.72	1.89	15.02	26.73	9.10	227	23.44	1.84	18.44	28.64	7.86
ADG	kg/d	902	401	0.14	0.06	-0.02	0.36	40.60	226	0.24	0.07	0.06	0.47	30.41
ETD	min	897	398	85.51	27.79	9.56	281.62	32.49	227	95.72	30.00	43.41	255.36	31.34
ETE	min	899	400	11.75	4.94	3.03	36.43	42.06	227	10.09	3.87	3.66	43.18	38.34
DNE	number	895	399	8.29	3.89	2.34	28.00	46.92	227	10.15	3.33	4.07	23.38	32.78
EFI	g of DM	902	400	294.52	105.19	37.70	600.45	35.72	227	286.15	77.79	132.71	639.30	27.19
ER	g/min	902	400	26.55	7.44	1.91	51.82	28.01	227	29.93	7.42	8.06	55.89	24.80

### Heritability, Genomic Prediction Accuracy and Bias

The heritabilities (h^2^ ± SE) of MeP, MeY and RMP were 0.18 ± 0.05, 0.13 ± 0.04 and 0.13 ± 0.04, respectively, (Table 5). The heritabilities of DMI and RFI were 0.34 ± 0.05 and 0.14 ± 0.04. MMWT and ADG had heritabilities of 0.46 ± 0.04 and 0.28 ± 0.05. The heritability of ETD and ETE were 0.29 ± 0.05 and 0.31 ± 0.05. DNE, EFI, and ER had heritabilities of 0.32 ± 0.05, 0.45 ± 0.04 and 0.47 ± 0.04. Genomic prediction accuracies were significant based on the standard errors and tended to be larger for traits with higher heritabilities (Table 4). For methane emission traits, the highest accuracy was obtained for MeY (0.30 ± 0.06), followed by RMP (0.13 ± 0.09), and MeP (0.12 ± 0.08). Genomic prediction accuracies for DMI and RFI were 0.16 ± 0.08 and 0.17 ± 0.07, and for MMWT and ADG they were 0.20 ± 0.06 and 0.27 ± 0.07, respectively. ETD and ETE had accuracies of 0.68 ± 0.03 and 0.40 ± 0.07, while the accuracies of DNE, EFI, and ER were 0.27 ± 0.09, 0.34 ± 0.08 and 0.41 ± 0.08, respectively. Except for MeP and MDI, the bias of all traits was close to one. Except for the methane emission traits and RFI, the standard error of the bias was relatively low (Table 4).

**TABLE 4:** Estimated heritabilities, genomic prediction accuracies and bias for methane emissions, production, maintenance and feeding behaviour traits in post-weaning, hogget, and adult Australian Maternal Composite sheep. * Methane production (MeP), methane yield (MeY), residual methane production (RMP), dry matter intake (DMI), residual feed intake (RFI), metabolic mid-weight (MMWT), average daily gain (ADG), eating time per day (ETD), eating time per eating event (ETE), daily number of events (DNE), event feed intake (EFI), and eating rate (ER).

Trait *	Unit	Heritability	Accuracy	Bias
MeP	L/d	0.18 ± 0.05	0.12 ± 0.08	0.76 ± 0.49
MeY	L/kg DMI	0.13 ± 0.04	0.30 ± 0.06	1.10 ± 0.58
RMP	L/d	0.13 ± 0.04	0.13 ± 0.09	0.82 ± 0.87
DMI	kg/d	0.34 ± 0.05	0.16 ± 0.08	0.79 ± 0.37
RFI	kg/d	0.14 ± 0.04	0.17 ± 0.07	1.02 ± 0.51
MMWT	kg	0.46 ± 0.04	0.20 ± 0.06	0.83 ± 0.28
ADG	kg/d	0.28 ± 0.05	0.27 ± 0.07	0.92 ± 0.22
ETD	min	0.29 ± 0.05	0.68 ± 0.03	0.99 ± 0.17
ETE	min	0.31 ± 0.05	0.40 ± 0.07	0.89 ± 0.24
DNE	number	0.32 ± 0.05	0.27 ± 0.09	0.83 ± 0.30
EFI	g of DM	0.45 ± 0.04	0.34 ± 0.08	0.96 ± 0.27
ER	g/min	0.47 ± 0.04	0.41 ± 0.08	0.95 ± 0.21

**FIGURE 2:**
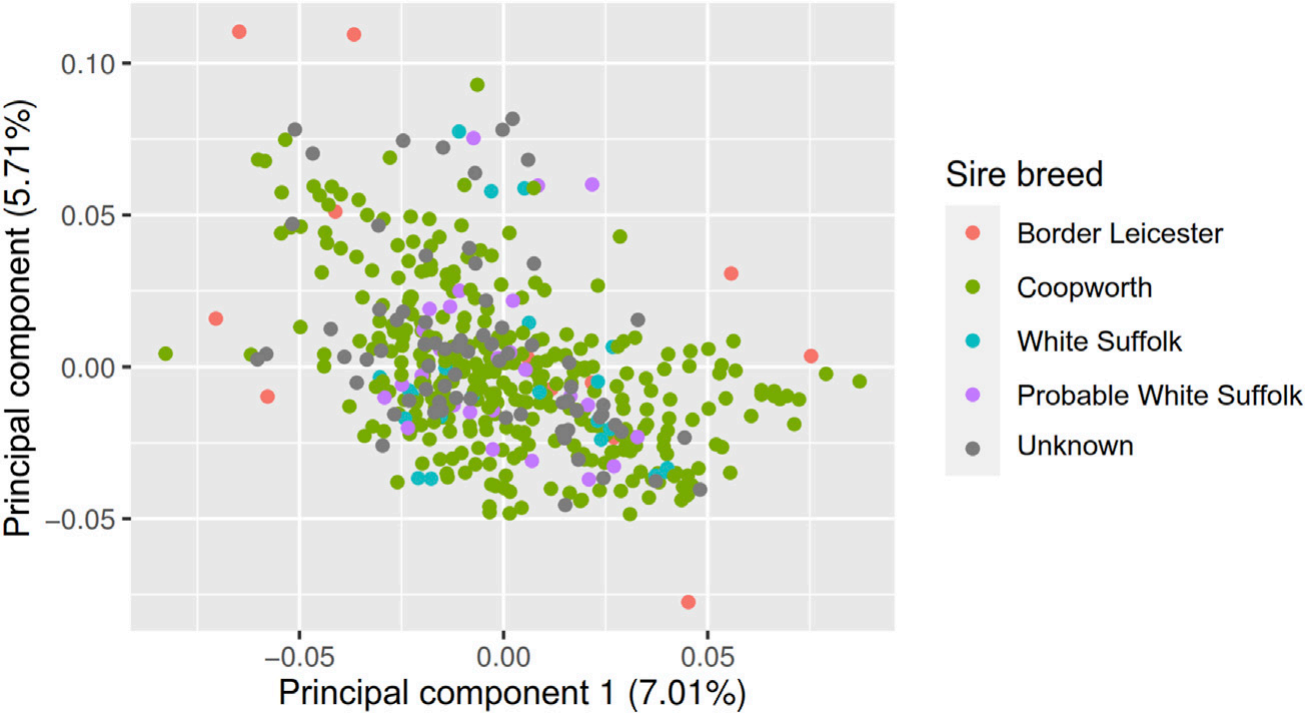
Principal component decompositions of genomic relationship matrix constructed from 38,378 SNP genotypes from post-weaning, hogget, and adult Australian Maternal Composite sheep with two principal components shown (PC1, PC2) and points labelled by sire breed.

The fixed effects EXP, PEN:EXP were significant for all traits; AGE was significant for all traits except for MeY, ETD, and ETE; Pregnancy was significant for MeP, MeY and MMWT; Pregnancy Scan was significant for MeP DMI, and MMWT (Table 5). The first two principal components of the **GRM** matrix together explained 12% of the variation, and after removing three genetically distant animals from the dataset, the **GRM** showed no clusters per sire breed (Figure 2). However, to reduce the potential effect of population structure on the prediction accuracy, we included the first two principal components (PC1 and PC2) of the **GRM** as covariates in the genomic prediction models. PC1 had a significant effect on MeY (*p* = 3.7e-02), DMI (5.6e-02) and RFI (*p* = 4.2e-04), and PC2 was significant for MeP (*p* = 5.8e-02) and MMWT (*p* = 3.1e-02) (Table 5).

**TABLE 5:** Significance of fixed effects in univariate genomic best linear unbiased prediction models that combine phenotypes of Australian Maternal Composite ewes at post-weaning, hogget, and adult ages. Significance obtained with the Wald test [Pr (Chisq)]. Traits: Methane production (MeP), methane yield (MeY), residual methane production (RMP), dry matter intake (DMI), residual feed intake (RFI), metabolic mid-weight (MMWT), average daily gain (ADG), eating time per day (ETD), eating time per eating event (ETE), daily number of events (DNE), event feed intake (EFI), and eating rate (ER). Fixed effects: Experiment (EXP), pen nested into experiment (PEN:EXP), days of age (AGE), days of pregnancy at the end of the experiment (Pregnancy), number of lambs in pregnancy scan (Pregnancy Scan), first (PC1) and second (PC2) principal components of the genomic relationship matrix.

	EXP	PEN:EXP	AGE	Pregnancy	Pregnancy Scan	PC1	PC2
MeP	<0.001	<0.01	<0.001	<0.01	<0.01		<0.01
MeY	<0.001	<0.05		<0.05		<0.05	
RMP							
DMI	<0.001	<0.05	<0.001		<0.01	<0.01	
RFI						<0.001	
MMWT	<0.001	<0.001	<0.001	<0.001	<0.05		<0.05
ADG	<0.001	<0.001	<0.001				
ETD	<0.001	<0.01					
ETE	<0.001	<0.001					
DNE	<0.001	<0.001	<0.001				
EFI	<0.001	<0.001	<0.001				
ER	<0.001	<0.01	<0.001				

### Correlations Between Ages

The genetic correlations for DMI obtained with **model 2** between PW and hogget, PW and adult, and hogget and adult were 0.84 ± 0.16, 0.76 ± 0.31, and 0.7 ± 0.20, respectively. Despite the large standard error of these genetic correlations, the high correlations suggest that DMI is genetically very similar at all ages. The phenotypic correlations for DMI were 0.32 ± 0.06, 0.30 ± 0.10, and 0.52 ± 0.08 between PW and hogget, PW and adult, and hogget and adult, respectively. The multivariate models between age stages for the rest of the traits did not converge, possibly due to the small number of animals in each stage.

### Feeding Behaviour, Methane Emissions and Residual Feed Intake

Longer ETE was genetically associated with higher MeP and MeY and more efficient animals (low RFI). The genetic correlation of ETE with MeP was 0.44 ± 0.32 and with MeY was 0.63 ± 0.22 (Table 6). The genetic and phenotypic correlations between ETE and RFI were −0.50 ± 0.19 and −0.17 ± 0.04. Genetic and phenotypic correlations between MeY and RFI were also negative (*r*_*g*_ = −0.79 ± 0.15; *r*_*p*_ = −0.46 ± 0.03) (Table 6). Further analyses are required to see the effect of selecting on ETE over RMP.

**TABLE 6:** Estimated genetic correlations [± standard error; above diagonal (-----)] and phenotypic correlations (± standard error; below diagonal) between traits in Australian Maternal Composite ewes estimated with bivariate GBLUP models of Maternal Composite ewes at post-weaning, hogget, and adult age stages. **Model 1** with fixed effects experiment, pen nested into experiment, age, pregnancy, number of lambs in pregnancy scan, and first two principal components of the genomic relationship matrix. **Model 2** with first two principal components of the genomic relationship matrix as fixed effects. **Model 2** used to obtain correlations between RFI and RMP and with their regressors, and when model 1 did not converge. Blank spaces are models that did not converge with any model. Traits: Methane production (MeP), methane yield (MeY), residual methane production (RMP), dry matter intake (DMI), residual feed intake (RFI), metabolic mid-weight (MMWT), average daily gain (ADG), eating time per day (ETD), eating time per eating event (ETE), daily number of events (DNE), event feed intake (EFI), and eating rate (ER).

**	MeP	MeY	RMP	DMI	RFI	MMWT	ADG	ETD	ETE	DNE	EFI	ER
MeP	-----	0.90 ± 0.14*	0.74 ± 0.13*	0.36 ± 0.26*	−0.01 ± 0.28*			0.36 ± 0.19	0.44 ± 0.32	−0.18 ± 0.27		0.05 ± 0.17
MeY	0.54 ± 0.02*	-----	0.80 ± 0.11	−0.43 ± 0.15	−0.79 ± 0.15	0.14 ± 0.19	0.18 ± 0.24*	0.07 ± 0.18	0.63 ± 0.22	−0.33 ± 0.15	0.31 ± 0.22*	−0.08 ± 0.20
RMP	0.60 ± 0.02*	0.84 ± 0.01	-----	−0.15 ± 0.30*	−0.26 ± 0.24*	0.13 ± 0.32*		0.04 ± 0.18	0.01 ± 0.18	−0.06 ± 0.19	−0.24 ± 0.21*	0.03 ± 0.19
DMI	0.67 ± 0.02*	−0.42 ± 0.03	0.01 ± 0.03*	-----	0.74 ± 0.14*	0.73 ± 0.16*			−0.13 ± 0.16	−0.06 ± 0.38*		−0.21 ± 0.25*
RFI	0.05 ± 0.03*	−0.46 ± 0.03	0.00 ± 0.03*	0.45 ± 0.03*	-----	0.33 ± 0.31*	0.34 ± 0.16	−0.12 ± 0.20*	−0.50 ± 0.19	0.43 ± 0.26*		0.45 ± 0.22*
MMWT		−0.12 ± 0.04	0.01 ± 0.03*	0.84 ± 0.01*	0.00 ± 0.03*	-----		0.13 ± 0.11	0.70 ± 0.27	0.41 ± 0.21		0.09 ± 0.12
ADG		0.24 ± 0.03*			0.00 ± 0.04		-----	0.24 ± 0.13		−0.44 ± 0.38*		0.25 ± 0.25*
ETD	−0.04 ± 0.04	−0.16 ± 0.04	−0.07 ± 0.04		0.30 ± 0.03*	−0.05 ± 0.04	0.16 ± 0.04	-----	0.59 ± 0.13*	0.55 ± 0.10	−0.34 ± 0.13*	−0.94 ± 0.05*
ETE	−0.32 ± 0.03	−0.11 ± 0.04	−0.08 ± 0.04	−0.18 ± 0.04	−0.17 ± 0.04	−0.21 ± 0.04		0.36 ± 0.03*	-----	−0.06 ± 0.26*		−0.07 ± 0.17
DNE	−0.12 ± 0.04	−0.18 ± 0.03	0.03 ± 0.04	0.01 ± 0.03*	0.29 ± 0.03*	−0.20 ± 0.04	0.33 ± 0.03*	0.36 ± 0.03	−0.58 ± 0.02*	-----	−0.57 ± 0.17*	−0.04 ± 0.19
EFI		−0.16 ± 0.03*	−0.05 ± 0.03*					−0.25 ± 0.03*		−0.73 ± 0.02*	-----	
ER	−0.04 ± 0.04	−0.24 ± 0.03	−0.04 ± 0.04	0.58 ± 0.02*	0.04 ± 0.04*	0.36 ± 0.03	−0.01 ± 0.03*	−0.61 ± 0.02*	−0.58 ± 0.02	−0.43 ± 0.03		-----

### Potential Response to Selection

**FIGURE 3:**
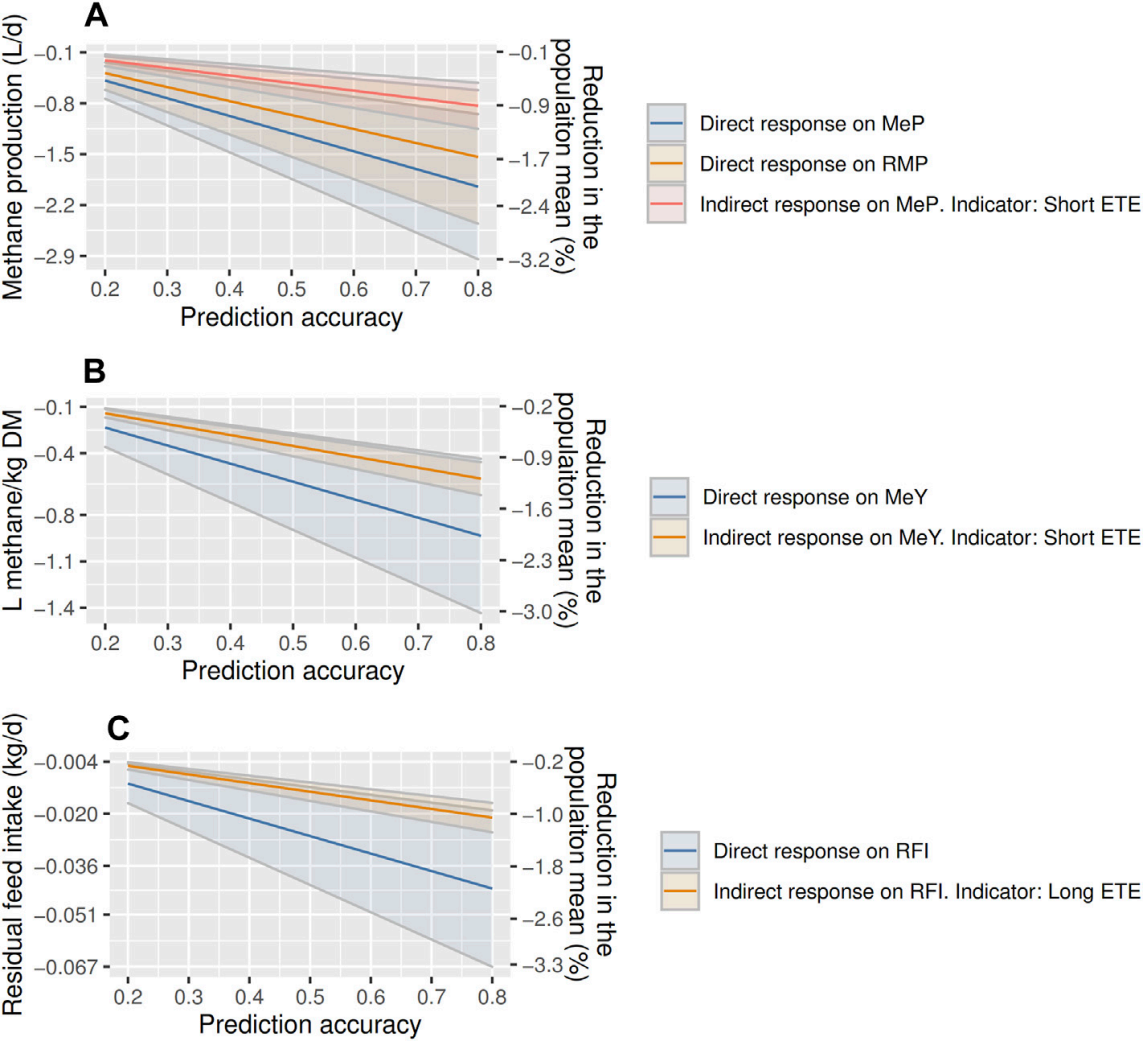
Annual response to selection on methane production [MeP; **(A)**], residual methane production [RMP; **(A)**], methane yield [MeY = MeP/DMI; **(B)**], and residual feed intake [RFI; **(C)**] using direct selection, or indirect selection with eating time per eating event (ETE) as indicator trait in post-weaning, hogget, and adult Australian Maternal Composite ewes. Solid lines indicated the estimated responses to selection, and the highlighted areas are the standard errors.

ETE could be used as indicator to reduce emissions by selecting for short ETE, and to reduce RFI by selecting for long ETE. Annual MeP is expected to decrease between ∼−0.2 and −2.9 L (−0.5 to −3.2%) with direct selection and between ∼−0.13 and −1.15 L (−0.5 to −1.0%) by indirectly selecting on shorter ETE (Figure 3). The annual decrease of RMP is projected between −0.2 and −2.5 L of CH_4_ (−0.5 to −2.8% of MeP) with direct selection. Yearly decreases in MeY are projected between −0.2 and −1.4 L/kg of DMI (−0.4 to −3.0%) with direct selection, and between −0.1 and −0.7 indirectly by selecting on shorter ETE (−0.2 to −1.5%; Figure 3). RFI is projected to decrease between −0.005 and −0.067 kg (−1.1–3.3%) with direct selection, and between −0.004 and −0.024 kg (−0.9–1.1%) indirectly by selecting on longer ETE (Figure 3).

## Discussion

Maternal Composite sheep are a composite used for ewe replacement in Australia, which has been mainly selected for improving fertility and size traits (Swan et al., 2017; Muir et al., 2020a). As they are a popular breed of sheep in Australia, it is of interest to investigate the breed’s genetic parameters and the potential for genomic selection of methane emissions and feed efficiency. Our results showed positive genetic correlations of ETE with methane emissions and a negative correlation between ETE and feed efficiency (positive with RFI), which suggests that ETE could potentially be evaluated as an indicator trait for emissions and feed efficiency in breeding programs.

The number of eating events per hour throughout the day-cycle was relatively constant in some of the animals we used when they were kept in the same facilities and under the same conditions as our study (Behrendt et al., 2021). Additionally, the sampling procedure we used was designed to minimise the effect of time of day on methane emissions. However, it is advisable for future studies to estimate the effect of feeding behaviour changes throughout the day on the heritability of and the correlations between methane emissions, feed efficiency, and the feeding behaviour traits.

There is potential to obtain ETE in large populations (including commercial farms) with the use of wearable sensor devices for measuring aspects of feeding behaviour for sheep (Fogarty et al., 2020), although it should be noted that these sorts of devices are still under development. If the ETE recorded by the automated feeding system we used is confirmed as the same trait as ETE recorded at pasture and if the correlations between ETE and methane emissions and RFI reported in our study are confirmed in grazing conditions, then ETE traits could be evaluated as indicative traits for reducing emissions and improving feed efficiency in grazing systems through wearable sensor devices.

### Heritability, Genomic Prediction Accuracy and Bias

The heritabilities reported in this manuscript agree with the literature (Table 1). The heritability of MeP and MeY obtained in this study were closer to those obtained with the same chambers we used (portable accumulation chamber) than with respiration chambers, probably because respiration chambers allow to obtain more accurate methane emissions measurement. This is the first paper that reports the heritability of RMP in sheep, but similar heritabilities have been reported in beef and dairy cattle. Our estimated heritability for RFI agrees with numerous other reports that included the same (or very similar) energy sinks, namely maintenance and growth—and is lower than literature estimates that are also corrected for body backfat and muscle depth (Table 1). Therefore, by including these body composition traits as RFI regressor in our study, a higher RFI heritability could be expected. Additionally, including body backfat and muscle depth as RFI regressors could improve the RFI breeding values prediction accuracy and obtain more accurate correlations (lower standard errors) between RFI and other traits and between RFI measured at different age stages. Therefore, it is advisable to include body compositions traits, as body backfat and muscle depth, as regressors of RFI in further analyses. The reported heritability estimates for DMI, ADG, and MMWT were in the range reported in other studies. The heritability of ETD was closer to other studies in sheep and lower than in dairy heifers, and ETE had a heritability close to those previously reported (Table 1). The heritabilities of DNE, EFI, and ER also agree with other research in sheep and dairy heifers.

The accuracies of the breeding values for methane emissions and RFI are not high enough, for genomic prediction. These lower accuracies could be do to the relatively small reference population and to the fact that we used a multibreed population. It has been estimated that to achieve a predicted accuracy of around 0.7 in two genetically distant populations of dairy cattle for traits with heritabilities similar to those we reported for methane emission traits and RFI (between 0.13 and 0.18), it is necessary to include close to 10,000 animals of each of these two population (20,000 in total) (Wientjes et al., 2016). This scenario gives an idea of the number of animals required to achieve higher accuracies in multibreed populations, as the Australian Maternal Composite sheep, for methane emission traits and RFI. Additionally, it is advisable that the animals selected for the reference population represent the variability of the population to avoid predictions biases. Accuracies of feeding behaviour traits tended to be higher than those in the rest of the traits, which is expected given their higher heritabilities. Biases of RFI, ADG, ETD, ETE, EFI, and ER were close to 1.0 with no large standard errors, suggesting they were well estimated. Prediction biases of methane emission traits had large standard errors, and no conclusions could be drawn about the bias. Biases of DMI, MMWT, and DNE were smaller than 1.0 with a relatively large standard error, suggesting they could potentially be overestimated.

### Correlations Between Ages

The genetic correlations for DMI between different ages were similar to those Paganoni et al. (2017) reported, between 0.64 and 0.78. It is advisable to increase the population size in further studies to estimate the genetic correlations between RFI, methane emissions, feeding behaviour, and related traits at different ages with minor standard errors and determine whether the traits are the same in all ages. However, previous studies have reported positive correlations between DMI, RFI, MeP, and body weight measured at different ages. Using some of the same animals in the present study, Muir et al. (2020a) reported phenotypic correlations between ages for DMI between 0.40 and 0.44, RFI between 0.17 and 0.20, and MeP between 0.20 and 0.24. The phenotypic correlations for DMI estimated by Muir et al. (2020a) are similar to ours, but not the same, possibly because we did not use exactly the same animals. Paganoni et al. (2017) reported stronger phenotypic correlations between the closest ages (PW-hogget and hogget-adult) than between PW and adults for feed intake, RFI and MeP. In the same study, the genetic correlations between ages for RFI had large standard errors, suggesting the necessity of obtaining data from larger datasets, which agrees with our results (Paganoni et al., 2017). In a study of up to 4,000 animals, MeP was reported as genetically the same trait in lambs and adults (*r*_*g*_ = 0.99) (Jonker et al., 2018b). Dominik and Oddy (2015) obtained repeatabilities for MeP measured in yearling and adult ewes between 0.17 and 0.27, which are similar to the phenotypic correlation (*r*_*p*_ = 0.21) reported by Jonker et al. (2018b). The correlations between MeP and body weight were consistent when they were analysed within and between ages (Jonker et al., 2018b).

### Potential Direct Response to Selection

The responses to selection using the GEBVs obtained with the data we used in this research are encouraging and agree with the reported literature. Rowe et al. (2019) reported a reduction of −12% for MeY in a decade (∼−1.2% per year) in a maternal sheep population, showing that breeding for lowered methane emissions is a permanent and cumulative strategy for reducing methane emissions in sheep. Similar percentages could be achieved in Australian Maternal Composite ewes by directly selecting for MeP (∼−2.0%), MeY (∼−2.0%), and RMP (∼−1.7% of MeP) assuming the realistic prediction accuracy of 0.3 (Figure 3). Using the same accuracy (0.3), smaller improvement were projected in beef cattle, −0.4% for MeP and −0.5% for MeY (Hayes et al., 2015). Selection for RMP would have a lower impact on MeP than the direct selection on MeP, but it would avoid potential deleterious effects on maintenance, growth, and feed intake. The direct response to selection of RFI in beef cattle was -0.5 kg of DMI in 6 years (−0.09 per year) (Arthur et al., 2001), which agrees with the −0.011 and −0.045 kg we calculated, taking into account the size difference between cattle and sheep.

### Eating Time as an Indicator Trait For Methane Emission and Feed Efficiency in Breeding Programs

In our study, the genetic correlations of RFI with MeY and RMP were negative. The negative correlation between RFI and MeY indicates that more efficient animals (low RFI) tend to emit more methane per kg of DMI. The negative correlation between RFI and RMP suggests that more efficient animals tend to emit more methane after adjusting for methane emissions expected to occur due to DMI, MMWT and ADG. This is the first paper that reports the genetic correlations between methane emissions and RFI in sheep fed pellets with high NDF content. Nutrition studies indicate that selecting low methane emitting sheep and cattle may result in reduced efficiency of cell wall digestion (that is NDF) (Løvendahl et al., 2018), which could lead to a decrease in feed efficiency for animals consuming high-fibre diets. This reduction of NDF digestion by selecting lower emitters could be present in our study but not as high as in grazing animals because pelleted diet and *ad libitum* intakes may favour shorter retention times of feed particles in the rumen, diminishing rumination, which is the primary digestion process involved in particle size reduction and digestion of cell walls.

The underlying biology for variation in methane emissions and feed intake in ruminants is still unknown because there are many interacting biological variables (Løvendahl et al., 2018). It is known that digesta kinetics influence methane emission in sheep (Pinares-Patiño et al., 2003a; Pinares-Patiño et al., 2003b; Pinares-Patiño et al., 2011a; Pinares-Patiño et al., 2011b; Goopy et al., 2014; Goopy et al., 2016), and ET could be playing an unknown role in these dynamics. Our results showed that longer ETE was genetically associated with higher MeP and MeY, and lower RFI (more efficient animals). Similarly, a negative correlation between RFI and ETE (−0.22) was previously reported in sheep, suggesting that more efficient animals tended to spend more time eating per eating event (Marie-Etancelin et al., 2019). Based on our results, we hypothesise that with the pelleted high NDF diet fed in our experiment, sheep that spend more time eating are more feed efficient because of a larger reduction of particle size (even when eating chewing does not reduce the particle size as much as rumination) and an increment in salivation, which would increase digestibility (decreasing RFI), but increase methane emissions due to the higher exposure of feed particles to methanogens. This situation would lead to an inverse relationship between RFI and methane emissions, i.e., more efficient animals produce more methane.

Longer eating time could imply more chewing and salivation whilst consuming feed, which increases feed digestibility. The bicarbonate and phosphate present in saliva maintain an optimum ruminal pH for microbial digestion through processes such as the bicarbonate-dependent absorption of volatile fatty acids (Beauchemin, 2018). Salivation is constant during chewing (Beauchemin, 2018), and longer eating time is expected to increase salivation. Additionally, it has been shown that salivation is higher in cattle fed with forages than concentrate (Beauchemin et al., 2008; Beauchemin, 2018). In turn, chewing increases digestibility by stimulating saliva secretion (which increases feed digestibility as previously mentioned) and breaking down the food to allow fungi access, bacteria adhesion, and biofilms formation that progressively degrade carbohydrates (Leng, 2014). It is advisable to validate, under similar dietary and environmental conditions to those we used, whether the relationships we have described between eating time, digestive processes, and methane emission differ between the higher and lower emitting sheep in our study and include rumination time and other relevant rumen functions in the analysis.

The negative correlation observed between RFI and MeY may be due to the high correlation of RFI with DMI which is in the denominator of MeY, and this implies an inverse relationship between MeY and RFI. Supporting this idea, the genetic correlation between DMI and MeY was also negative in our study. Another possible explanation for the negative correlation between RFI and MeY that is mentioned in Flay et al. (2019) is that low RFI animals produce similar MeP to high RFI animals but ate less (lower DMI), making low RFI individuals produce more MeY (MeP/DMI). Another potential reason for the negative genetic correlations between RFI with MeY that also could explain the negative correlation between RFI and RMP is that under the relatively high-NDF diet used in our experiment, animals that spent more time eating are more feed efficient because of a higher chewing and salivation, which would increase digestibility as previously discussed, and methane emissions would increase a consequence of a higher exposure of feed particles to methanogens in the rumen. Comparing the digestion kinetics of higher and lower emitters would allow to confirm or discard this hypothesis.

There could be a genotype × diet interaction in relationships between eating time, methane emissions and feed efficiency. Low-RFI beef cows produced less MeP under high-quality pasture, but not under a low-quality pasture (Jones et al., 2011). It was reported in beef steers that MeP and MeY increases when forage is supplied and supplementation is reduced (Wallace et al., 2014). In contrast, low MeY selection line sheep emitted lower MeY regardless of the NDF content of two pastures (Jonker et al., 2018a). In concordance with our results, lower RFI heifers fed with alfalfa cubes had higher MeY (Flay et al., 2019). In contrast to our result, but feeding pellets with lower NDF-content (34.6%), Paganoni et al. (2017) reported that more efficient hoggets emit less methane (r_g_RFI-MeP = 0.76; r_g_RFI-MeY = 0.46). Feed particle size could influence the correlations between emissions, RFI, and ET. Longer ET may have reduced the particle size of the pellets, leading to feed efficiency improvement, and increasing exposure to digestion and therefore producing more methane. The presentation of pellets in a trough to sheep for consumption is different to that of intact forages where the ability of sheep to consume feed and the amount of time spent grazing is strongly influenced by the quality, height and density of the forage and the bite size and bite rate that can be achieved (Allden and Mcdwhittaker, 1970). Therefore, further studies investigating eating time in relation to methane emissions and RFI of sheep under grazing systems is advisable.

If ETE is confirmed as an indicator trait for RFI and methane emissions in research facilities, then the next step would be an evaluation in larger populations (including commercial farms) with the use of devices that monitor feeding. Those devices are a promising technology for the sheep industry (Fogarty et al., 2020) that is already commercially available in cattle at a relatively low cost (Beauchemin, 2018). Using devices that can measure aspects of feeding behaviour in large populations that are also genotyped, would enable higher genomic prediction accuracy for ETE compared with RFI and methane emission traits that are time-consuming and expensive to measure, or require specialist equipment. Additionally, it is advisable for further studies to investigate the effect of eating time on the number and size of eating events, as changes in these traits could have implications for feeding management.

The correlations between ETD and RFI should be evaluated in a larger reference population. A larger population is also needed to accurately estimate the genetic correlations of RMP with ETD and ETE. These unknown genetic correlations would help to elucidate the effect that selecting for eating time would have on emissions and feed efficiency. Studies at pasture are required to confirm our results and their application in extensive grazing systems such as those practice in Australia. If the correlations found here are confirmed, ETE could be used as an indicator trait for methane emissions and RFI, but an index approach would be required to simultaneously select for benefits in emissions, RFI, and related traits. Combinations of indicator traits appear to provide the most accurate estimates of methane emissions (Negussie et al., 2017), and based on our results, ETE may also improve accuracy.

## Conclusion

This study presented results on the genetic basis of methane emissions, feed intake, eating time, and related traits in sheep. As expected, prediction accuracies tend to increase proportionally to the heritability of the trait. Expansion of training sets and recording the traits under grazing conditions is required to achieve accurate predictions before these traits can be included in breeding programs for sheep kept on pasture.

In our data, selecting for more efficient animals (low RFI) would lead to higher methane emissions per day and per kg of dry matter intake. In the same way, selecting for lower emitters per day or kg of dry matter intake would lead to less efficient animals (high RFI). Selecting for longer eating time per eating event would improve feed efficiency but would also increase the methane emissions per day and per kg of dry matter intake. Therefore, an index approach would be needed to select simultaneously for benefits in both methane emissions and feed efficiency, either by direct selection or by indirect selection using eating time per eating event as indicator trait. Further analyses in larger reference population and in grazing systems are advised to confirm the association between eating time, methane emissions, and RFI. If these associations are confirmed, selecting for eating time traits could have a tremendous impact on the sheep industry.

## Data Availability

The datasets presented in this article are not readily available because these are the exclusive property of the Department Of Jobs Precincts And Regions (DJPR), Victoria, Australia. Requests to access the datasets should be directed to Dr Matthew Knight, Research Manager, matthew.knight@agriculture.vic.gov.au.

## References

[B1] Allden W. Mcdwhittaker I. (1970). The Determinants of Herbage Intake by Grazing Sheep: The Interrelationship of Factors Influencing Herbage Intake and Availability. Aust. J. Agric. Res. 21, 755. 10.1071/ar9700755

[B2] Amadeu R. R. Cellon C. Olmstead J. W. Garcia A. A. F. Resende M. F. R. Muñoz P. R. (2016). AGHmatrix: R Package to Construct Relationship Matrices for Autotetraploid and Diploid Species: A Blueberry Example. Plant Genome 9, 1–10. 10.3835/plantgenome2016.01.0009 27902800

[B3] Amarilho-Silveira F. de Barbieri I. Cobuci J. A. Balconi C. M. de Ferreira G. F. Ciappesoni G. (2022). Residual Feed Intake for Australian Merino Sheep Estimated in Less Than 42 Days of Trial. Livest. Sci. 258, 104889. 10.1016/j.livsci.2022.104889

[B4] Arthur P. R. Archer J. A. Herd R. M. Melville G. J. (2001). “Response to Selection for Net Feed Intake in Beef Cattle,” in Proc. Assoc. Advmt. Anim. Breed. Genet. Queenstown, New Zealand 14, 135–138.

[B5] Beauchemin K. A. Eriksen L. Nørgaard P. Rode L. M. (2008). Short Communication: Salivary Secretion during Meals in Lactating Dairy Cattle. J. Dairy Sci. 91, 2077–2081. 10.3168/jds.2007-0726 18420637

[B6] Beauchemin K. A. (2018). Invited Review: Current Perspectives on Eating and Rumination Activity in Dairy Cows. J. Dairy Sci. 101, 4762–4784. 10.3168/jds.2017-13706 29627250

[B7] Behrendt R. Muir S. K. Moniruzzaman M. Kearney G. Knight M. I. (2021). Automated Feeding of Sheep. 1. Changes in Feeding Behaviour in Response to Restricted and *Ad Libitum* Feeding. Anim. Prod. Sci. 61, 246–255. 10.1071/AN20146

[B8] Bibé B. O. Brunel J. C. Bourdillon Y. Loradoux D. Gordy M. H. Weisbecker J. L. (2002). “Genetic Parameters of Growth and Carcass Quality of Lambs at the French Progeny-Test Station Berrytest,” in Proc. 7th World Cong. Genet. Appl. Livest. Prod. (Montpellier, France: World Congress on Genetics Applied to Livestock Production (WCGALP)).

[B9] Bolormaa S. Chamberlain A. J. Khansefid M. Stothard P. Swan A. A. Mason B. (2019). Accuracy of Imputation to Whole-Genome Sequence in Sheep. Genet. Sel. Evol. 51, 1. 10.1186/s12711-018-0443-5 30654735 PMC6337865

[B10] Bolormaa S. Pryce J. E. Kemper K. Savin K. Hayes B. J. Barendse W. (2013). Accuracy of Prediction of Genomic Breeding Values for Residual Feed Intake and Carcass and Meat Quality Traits in *Bos taurus*, Bos indicus, and Composite Beef Cattle1. J. Anim. Sci. 91, 3088–3104. 10.2527/jas.2012-5827 23658330

[B11] Butler D. Cullis B. R. Gilmour A. Gogel B. (2009). ASReml-R Reference Manual. Queensland: Department of Primary Industries and Fisheries.

[B12] Cammack K. M. Leymaster K. A. Jenkins T. G. Nielsen M. K. (2005). Estimates of Genetic Parameters for Feed Intake, Feeding Behavior, and Daily Gain in Composite Ram Lambs1,2. J. Anim. Sci. 83, 777–785. 10.2527/2005.834777x 15753331

[B13] Daetwyler H. D. Kemper K. E. Van Der Werf J. H. J. Hayes B. J. (2012). Components of the Accuracy of Genomic Prediction in a Multi-Breed Sheep Population1. J. Anim. Sci. 90, 3375–3384. 10.2527/jas.2011-4557 23038744

[B14] de Haas Y. Pryce J. E. Wall E. McParland S. Manzanilla Pech C. I. V. Difford G. (2016). 0407 Genomic Selection for Methane Emission. J. Anim. Sci. 94, 197–198. 10.2527/jam2016-0407

[B15] de Haas Y. Pszczola M. Soyeurt H. Wall E. Lassen J. (2017). Invited Review: Phenotypes to Genetically Reduce Greenhouse Gas Emissions in Dairying. J. Dairy Sci. 100, 855–870. 10.3168/jds.2016-11246 27939541

[B16] Dominik S. Oddy V. (2015). “Repeatabilities for Methane Emission in Merino Ewes on Pasture across Different Ages,” in Proc. Assoc. Advmt. Anim. Breed. Genet. Lorne, VIC 21, 110–113.

[B17] Flay H. E. Kuhn-Sherlock B. Macdonald K. A. Camara M. Lopez-Villalobos N. Donaghy D. J. (2019). Hot Topic: Selecting Cattle for Low Residual Feed Intake Did Not Affect Daily Methane Production but Increased Methane Yield. J. Dairy Sci. 102, 2708–2713. 10.3168/jds.2018-15234 30639015

[B18] Fogarty E. S. Swain D. L. Cronin G. M. Moraes L. E. Trotter M. (2020). Behaviour Classification of Extensively Grazed Sheep Using Machine Learning. Comput. Electron. Agric. 169, 105175. 10.1016/j.compag.2019.105175

[B19] Fogarty N. M. (1995). Genetic Parameters for Live Weight, Fat and Muscle Measurements, Wool Production and Reproduction in Sheep: a Review. Anim. Breed. Abstr. 63, 101–143.

[B20] Fogarty N. M. Lee G. J. Ingham V. M. Gaunt G. M. Cummins L. J. (2006). Variation in Feed Intake of Grazing Crossbred Ewes and Genetic Correlations with Production Traits. Aust. J. Agric. Res. 57, 1037. 10.1071/ar05403

[B21] Fogarty N. M. Safari E. Mortimer S. I. Greeff J. C. Hatcher S. (2009). Heritability of Feed Intake in Grazing Merino Ewes and the Genetic Relationships with Production Traits. Anim. Prod. Sci. 49, 1080. 10.1071/an09075

[B22] François D. Bibé B. Bouix J. Brunel J. Weisbecker J. Ricard E. (2002). “Genetic Parameters of Feeding Traits in Meat Sheep,” in Proc. 7th World Cong. Genet. Appl. Livest. Prod. (Montpellier, France: INRA).

[B23] Goopy J. P. Donaldson A. Hegarty R. Vercoe P. E. Haynes F. Barnett M. (2014). Low-methane Yield Sheep Have Smaller Rumens and Shorter Rumen Retention Time. Br. J. Nutr. 111, 578–585. 10.1017/s0007114513002936 24103253

[B24] Goopy J. P. Robinson D. L. Woodgate R. T. Donaldson A. J. Oddy V. H. Vercoe P. E. (2016). Estimates of Repeatability and Heritability of Methane Production in Sheep Using Portable Accumulation Chambers. Anim. Prod. Sci. 56, 116. 10.1071/an13370

[B25] Goopy J. P. Woodgate R. Donaldson A. Robinson D. L. Hegarty R. S. (2011). Validation of a Short-Term Methane Measurement Using Portable Static Chambers to Estimate Daily Methane Production in Sheep. Animal Feed Sci. Technol. 166-167, 219–226. 10.1016/j.anifeedsci.2011.04.012

[B26] Hayes B. J. Donoghue K. A. Reich C. Mason B. Herd R. M. Arthur P. F. (2015). “Genomic Estimated Breeding Values for Methane Production in Australian Beef Cattle,” in Proc. 21th Assoc. Advmt. Anim. Breed. Genet. (Lorne, Victoria: Association for the Advancement of Animal Breeding and Genetics (AAABG)), 118–121.

[B27] Herd R. M. Arthur P. F. Bird S. H. Donoghue K. A. Hegarty R. S. (2014). “Genetic Variation for Methane Traits in Beef Cattle,” in Proceedings, 10th World Congress of Genetics Applied to Livestock Production. Vancouver, Canada: World Congress of Genetics Applied to Livestock Production (WCGALP). 448**,** 253.

[B28] Hess M. K. Johnson P. L. Knowler K. Hickey S. M. Hess A. S. McEwan J. C. (2019). “GWAS for Methane Yield, Residual Feed Intake and Liveweight in New Zealand Sheep,” in Proc. 23th Assoc. Advmt. Anim. Breed. Genet. (Armidale: Association for the Advancement of Animal Breeding and Genetics (AAABG)), 302–305.

[B29] Johnson P. L. Wing J. Knowler K. Johnstone P. (2017). “Investigating Variation in the Test Length Required to Estimate the Trait of Residual Energy Intake in Growing Maternal Lambs,” in Proc. 22th Assoc. Advmt. Anim. Breed. Genet. (Townsville, Queensland, Australia: AAABG), 325–328.

[B30] Jones F. M. Phillips F. A. Naylor T. Mercer N. B. (2011). Methane Emissions from Grazing Angus Beef Cows Selected for Divergent Residual Feed Intake. Animal Feed Sci. Technol. 166-167, 302–307. 10.1016/j.anifeedsci.2011.04.020

[B31] Jonker A. Hickey S. McEwan J. C. Rowe S. Aharoni Y. Molano G. (2018a). “Rumen Characteristics and Total Tract Digestibility in Low and High Methane Yield Selection Line Sheep Offered Fresh Good or Poor Quality Pasture,” in Proceedings of the World Congress on Genetics Applied to Livestock Production. Vancouver, Canada: World Congress of Genetics Applied to Livestock Production (WCGALP).

[B32] Jonker A. Hickey S. McEwan J. Waghorn G. (2020). “Chapter 6: Portable Accumulation Chambers for Enteric Methane Determination in Sheep,” in Guidelines for Estimating Methane Emissions from Individual Ruminants Using: GreenFeed, 'sniffers', Hand-Held Laser Detector and Portable Accumulation Chambers. Wellington, New Zealand: Ministry for Primary Industries, New Zealand, 55–62.

[B33] Jonker A. Hickey S. M. Mcewan J. C. Rowe S. J. Janssen P. H. Maclean S. (2019). Genetic Parameters of Plasma and Ruminal Volatile Fatty Acids in Sheep Fed Alfalfa Pellets and Genetic Correlations with Enteric Methane Emissions1. J. Anim. Sci. 97, 2711–2724. 10.1093/jas/skz162 31212318 PMC6606511

[B34] Jonker A. Hickey S. M. Rowe S. J. Janssen P. H. Shackell G. H. Elmes S. (2018b). Genetic Parameters of Methane Emissions Determined Using Portable Accumulation Chambers in Lambs and Ewes Grazing Pasture and Genetic Correlations with Emissions Determined in Respiration Chambers1. J. Anim. Sci. 96, 3031–3042. 10.1093/jas/sky187 29741677 PMC6095386

[B35] Koch R. M. Swiger L. A. Chambers D. Gregory K. E. (1963). Efficiency of Feed Use in Beef Cattle. J. Anim. Sci. 22, 486–494. 10.2527/jas1963.222486x

[B36] Leng R. A. (2014). Interactions between Microbial Consortia in Biofilms: a Paradigm Shift in Rumen Microbial Ecology and Enteric Methane Mitigation. Anim. Prod. Sci. 54, 519. 10.1071/an13381

[B37] Leymaster K. A. Cammack K. M. Nielsen M. K. Jenkins T. G. (2002). “Estimates of Genetic Parameters for Daily Gain, Feed Intake, and Behavior Traits in Ram Lambs of a Composite Population,” in Proc. 7th World Cong. Genet. Appl. Livest. Prod. (Montpellier, France).

[B38] Li B. VanRaden P. M. Guduk E. O'Connell J. R. Null D. J. Connor E. E. (2020). Genomic Prediction of Residual Feed Intake in US Holstein Dairy Cattle. J. Dairy Sci. 103, 2477–2486. 10.3168/jds.2019-17332 31954583

[B39] Lin Z. Macleod I. Pryce J. E. (2013). Short Communication: Estimation of Genetic Parameters for Residual Feed Intake and Feeding Behavior Traits in Dairy Heifers. J. Dairy Sci. 96, 2654–2656. 10.3168/jds.2012-6134 23462165

[B40] Løvendahl P. Difford G. F. Li B. Chagunda M. G. G. Huhtanen P. Lidauer M. H. (2018). Review: Selecting for Improved Feed Efficiency and Reduced Methane Emissions in Dairy Cattle. Animal 12, s336–s349. 10.1017/s1751731118002276 30255826

[B41] Macleay C. Blumer S. Hancock S. Inglis L. Paganoni B. Rose G. (2016). “Feed Intake for Sheep Can Be Measured Precisely in Less Than 35 Days,” in Proc. 31th Aust. Soc. of Anim. Prod. (Adelaide, South Australia: Australian Society of Animal Production).

[B42] Manzanilla-Pech C. I. V. De Haas Y. Hayes B. J. Veerkamp R. F. Khansefid M. Donoghue K. A. (2016). Genomewide Association Study of Methane Emissions in Angus Beef Cattle with Validation in Dairy Cattle1. J. Anim. Sci. 94, 4151–4166. 10.2527/jas.2016-0431 27898855

[B43] Manzanilla-Pech C. I. V. L⊘vendahl P. Mansan Gordo D. Difford G. F. Pryce J. E. Schenkel F. (2021). Breeding for Reduced Methane Emission and Feed-Efficient Holstein Cows: An International Response. J. Dairy Sci. 104, 8983–9001. 10.3168/jds.2020-19889 34001361

[B44] Marie‐Etancelin C. Francois D. Weisbecker J. L. Marcon D. Moreno‐Romieux C. Bouvier F. (2019). Detailed Genetic Analysis of Feeding Behaviour in Romane Lambs and Links with Residual Feed Intake. J. Anim. Breed. Genet. 136, 174–182. 10.1111/jbg.12392 30945778

[B45] Mousa E. Van Vleck L. D. Leymaster K. A. (1999). Genetic Parameters for Growth Traits for a Composite Terminal Sire Breed of Sheep. J. Anim. Sci. 77, 1659–1665. 10.2527/1999.7771659x 10438010

[B46] Muir S. K. Behrendt R. Moniruzzaman M. Kearney G. Knight M. I. (2022). Automated Feeding of Sheep. 2. Feeding Behaviour Influences the Methane Emissions of Sheep Offered Restricted Diets. Anim. Prod. Sci. 62, 55. 10.1071/AN20634

[B47] Muir S. K. Linden N. Kennedy A. Knight M. I. Paganoni B. Kearney G. (2020a). Correlations between Feed Intake, Residual Feed Intake and Methane Emissions in Maternal Composite Ewes at Post Weaning, Hogget and Adult Ages. Small Ruminant Res. 192, 106241. 10.1016/j.smallrumres.2020.106241

[B48] Muir S. K. Linden N. Knight M. Behrendt R. Kearney G. (2018). Sheep Residual Feed Intake and Feeding Behaviour: Are 'nibblers' or 'binge Eaters' More Efficient? Anim. Prod. Sci. 58, 1459–1464. 10.1071/an17770

[B49] Muir S. K. Linden N. P. Kennedy A. Calder G. Kearney G. Roberts R. (2020b). Technical Note: Validation of an Automated Feeding System for Measuring Individual Animal Feed Intake in Sheep Housed in Groups. Transl. Anim. Sci. 4, 1006–1016. 10.1093/tas/txaa007 PMC7200410 32705008

[B50] NDC (2020). Australia’s Nationally Determined Contribution - Communication 2020. Australia: Australian Government.

[B74] Negussie E. De Haas Y. Dehareng F. Dewhurst R. J. Dijkstra J. Gengler N. (2017). Invited Review: Large-Scale Indirect Measurements for Enteric Methane Emissions in Dairy Cattle: A Review of Proxies and Their Potential for Use in Management and Breeding Decisions. J. Dairy Sci. 100, 2433–2453. 10.3168/jds.2016-12030 28161178

[B52] Paganoni B. Rose G. Macleay C. Jones C. Brown D. J. Kearney G. (2017). More Feed Efficient Sheep Produce Less Methane and Carbon Dioxide when Eating High-Quality Pellets. J. Anim. Sci. 95, 3839–3850. 10.2527/jas.2017.1499 28992015

[B53] Pinares-Patiño C. S. Ebrahimi S. McEwan J. Dodds K. Clark H. Luo D. (2011a). “Is Rumen Retention Time Implicated in Sheep Differences in Methane Emission?” in Proc. 71th New Zealand Society of Animal Production (Invercargill: New Zealand Society of Animal Production), 219–222.

[B54] Pinares-Patiño C. S. Hickey S. M. Young E. A. Dodds K. G. MacLean S. Molano G. (2013). Heritability Estimates of Methane Emissions from Sheep. Animal 7, 316–321. 10.1017/S1751731113000864 PMC3691003 23739473

[B55] Pinares-Patiño C. S. Mcewan J. C. Dodds K. G. Cárdenas E. A. Hegarty R. S. Koolaard J. P. (2011b). Repeatability of Methane Emissions from Sheep. Animal Feed Sci. Technol. 166-167, 210–218. 10.1016/j.anifeedsci.2011.04.068

[B56] Pinares-patiño C. S. Ulyatt M. J. Lassey K. R. Barry T. N. Holmes C. W. (2003a). Persistence of Differences between Sheep in Methane Emission under Generous Grazing Conditions. J. Agric. Sci. 140, 227–233. 10.1017/s0021859603003071

[B57] Pinares-patiño C. S. Ulyatt M. J. Lassey K. R. Barry T. N. Holmes C. W. (2003b). Rumen Function and Digestion Parameters Associated with Differences between Sheep in Methane Emissions when Fed Chaffed Lucerne Hay. J. Agric. Sci. 140, 205–214. 10.1017/s0021859603003046

[B58] Pryce J. E. Gonzalez-Recio O. Nieuwhof G. Wales W. J. Coffey M. P. Hayes B. J. (2015). Hot Topic: Definition and Implementation of a Breeding Value for Feed Efficiency in Dairy Cows. J. Dairy Sci. 98, 7340–7350. 10.3168/jds.2015-9621 26254533

[B59] Robinson D. L. Goopy J. P. Hegarty R. S. Vercoe P. E. (2010). “Repeatability, Animal and Sire Variation in 1-hr Methane Emissions & Relationships with Rumen Volatile Fatty Acid Concentrations,” in Proc. 9th World Cong. Genet. Appl. Livest. Prod. Leipzig, Germany: World Congress of Genetics Applied to Livestock Production (WCGALP).

[B60] Rowe S. J. Hickey S. M. Jonker A. Hess M. K. Janssen P. Johnson T. (2019). “Selection for Divergent Methane Yield in New Zealand Sheep - A Ten Year Perspective,” in Proc. 23th Assoc. Advmt. Anim. Breed. Genet. (Armidale, Australia: Association for the Advancement of Animal Breeding and Genetics (AAABG)).

[B61] Rowe S. J. McEwan J. C. Hickey S. M. Anderson R. A. Hyndman D. Young E. A. (2014). “Genomic Selection as a Tool to Decrease Greenhouse Gas Emission from Dual Purpose New Zealand Sheep,” in Proc. 10th World Cong. Genet. Appl. Livest. Prod. (Vancouver: World Congress on Genetics Applied to Livestock Production (WCGALP)).

[B62] Sargolzaei M. Chesnais J. P. Schenkel F. S. (2014). A New Approach for Efficient Genotype Imputation Using Information from Relatives. BMC Genomics 15, 478. 10.1186/1471-2164-15-478 24935670 PMC4076979

[B63] Saunois M. Stavert A. R. Poulter B. Bousquet P. Canadell J. G. Jackson R. B. (2019). The Global Methane Budget 2000-2017. Earth Syst. Sci. Data Discuss. 12, 1–138. 10.5194/essd-2019-128

[B64] Shrestha J. N. B. Vesely J. A. Chesnais J. P. Cuthbertson D. (1986). Genetic and Phenotypic Parameters for Daily Gain and Body Weights in Dorset Lambs. Can. J. Anim. Sci. 66, 289–292. 10.4141/cjas86-029

[B65] Shrestha J. N. B. Vesely J. A. Chesnais J. P. (1985). Genetic and Phenotypic Parameters for Daily Gain and Body Weights in Suffolk Lambs. Can. J. Anim. Sci. 65, 575–582. 10.4141/cjas85-068

[B66] Silva R. M. O. Fragomeni B. O. Lourenco D. A. L. Magalhães A. F. B. Irano N. Carvalheiro R. (2016). Accuracies of Genomic Prediction of Feed Efficiency Traits Using Different Prediction and Validation Methods in an Experimental Nelore Cattle Population1. J. Anim. Sci. 94, 3613–3623. 10.2527/jas.2016-0401 27898889

[B67] Snowder G. D. Van Vleck L. D. (2003). Estimates of Genetic Parameters and Selection Strategies to Improve the Economic Efficiency of Postweaning Growth in Lambs1. J. Anim. Sci. 81, 2704–2713. 10.2527/2003.81112704x 14601873

[B68] Swan A. A. Banks R. G. Brown D. J. Chandler H. R. (2017). “An Update on Genetic Progress in the Australian Sheep Industry,” in Proc. 22th Assoc. Advmt. Anim. Breed. Genet. (Townsville, Queensland, Australia: AAABG), 365–368.

[B69] Tortereau F. Marie-Etancelin C. Weisbecker J.-L. Marcon D. Bouvier F. Moreno-Romieux C. (2020). Genetic Parameters for Feed Efficiency in Romane Rams and Responses to Single-Generation Selection. animal 14, 681–687. 10.1017/S1751731119002544 31640830

[B70] VanRaden P. M. (2008). Efficient Methods to Compute Genomic Predictions. J. Dairy Sci. 91, 4414–4423. 10.3168/jds.2007-0980 18946147

[B71] Wallace R. J. Rooke J. A. Duthie C.-A. Hyslop J. J. Ross D. W. Mckain N. (2014). Archaeal Abundance in *Post-mortem* Ruminal Digesta May Help Predict Methane Emissions from Beef Cattle. Sci. Rep. 4, 5892. 10.1038/srep05892 25081098 PMC5376199

[B72] Wientjes Y. C. J. Bijma P. Veerkamp R. F. Calus M. P. L. (2016). An Equation to Predict the Accuracy of Genomic Values by Combining Data from Multiple Traits, Populations, or Environments. Genetics 202, 799–823. 10.1534/genetics.115.183269 26637542 PMC4788251

[B73] Yang J. Benyamin B. McEvoy B. P. Gordon S. Henders A. K. Nyholt D. R. (2010). Common SNPs Explain a Large Proportion of the Heritability for Human Height. Nat. Genet. 42, 565–569. 10.1038/ng.608 20562875 PMC3232052

